# *In Silico* Identification and *In Vitro* and *In Vivo* Validation of Anti-Psychotic Drug Fluspirilene as a Potential CDK2 Inhibitor and a Candidate Anti-Cancer Drug

**DOI:** 10.1371/journal.pone.0132072

**Published:** 2015-07-06

**Authors:** Xi-Nan Shi, Hongjian Li, Hong Yao, Xu Liu, Ling Li, Kwong-Sak Leung, Hsiang-fu Kung, Di Lu, Man-Hon Wong, Marie Chia-mi Lin

**Affiliations:** 1 Biotechnology Center, Kunming Medical University, Kunming, Yunnan, China; 2 Department of Computer Science and Engineering, Chinese University of Hong Kong, Hong Kong, China; 3 Department of Medicine, Southwest Guizhou Vocational and Technical College for Nationalities, Guizhou, China; 4 Shenzhen Key Lab of Translational Medicine of Tumor, School of Medicine, Shenzhen University, Shenzhen, China; 5 The Cancer Biotherapy Institute of Jiangsu Province, Xuzhou Medical College, Xuzhou, China; 6 School of Biomedical Sciences, Chinese University of Hong Kong, Hong Kong, China; Taipei Medicine University, TAIWAN

## Abstract

Hepatocellular carcinoma (HCC) is one of the leading causes of cancer-related deaths worldwide. Surgical resection and conventional chemotherapy and radiotherapy ultimately fail due to tumor recurrence and HCC’s resistance. The development of novel therapies against HCC is thus urgently required. The cyclin-dependent kinase (CDK) pathways are important and well-established targets for cancer treatment. In particular, CDK2 is a key factor regulating the cell cycle G1 to S transition and a hallmark for cancers. In this study, we utilized our free and open-source protein-ligand docking software, idock, prospectively to identify potential CDK2 inhibitors from 4,311 FDA-approved small molecule drugs using a repurposing strategy and an ensemble docking methodology. Sorted by average idock score, nine compounds were purchased and tested *in vitro*. Among them, the anti-psychotic drug fluspirilene exhibited the highest anti-proliferative effect in human hepatocellular carcinoma HepG2 and Huh7 cells. We demonstrated for the first time that fluspirilene treatment significantly increased the percentage of cells in G1 phase, and decreased the expressions of CDK2, cyclin E and Rb, as well as the phosphorylations of CDK2 on Thr160 and Rb on Ser795. We also examined the anti-cancer effect of fluspirilene *in vivo* in BALB/C nude mice subcutaneously xenografted with human hepatocellular carcinoma Huh7 cells. Our results showed that oral fluspirilene treatment significantly inhibited tumor growth. Fluspirilene (15 mg/kg) exhibited strong anti-tumor activity, comparable to that of the leading cancer drug 5-fluorouracil (10 mg/kg). Moreover, the cocktail treatment with fluspirilene and 5-fluorouracil exhibited the highest therapeutic effect. These results suggested for the first time that fluspirilene is a potential CDK2 inhibitor and a candidate anti-cancer drug for the treatment of human hepatocellular carcinoma. In view of the fact that fluspirilene has a long history of safe human use, our discovery of fluspirilene as a potential anti-HCC drug may present an immediately applicable clinical therapy.

## Introduction

Hepatocellular carcinoma (HCC) is the most common type of liver cancer. Only 30% to 40% of the HCC patients are eligible for curative treatments, which include surgical resection as the first option, liver transplantation and percutaneous ablation. However, there is a high frequency of tumor recurrence after surgical resection, and most HCCs seem resistant to conventional chemotherapy and radiotherapy. Therefore the development of novel therapies against HCC is highly demanded.

The cause of HCC involves multiple pathways. The cyclin-dependent kinase (CDK) pathways as important therapeutic targets for cancer treatment have been well established. CDKs are enzymes implicated in cell replication, and their role in tumor growth has long made them into attractive drug targets. But early industrial attempts at inhibiting CDKs to restore cell growth to normal have encountered toxicity issues. First-generation CDK inhibitors were non-specific, inhibiting many different CDKs (there are more than 20, many of which have been implicated in various tumor types), and resulting in the type of toxicities and muted efficacy seen with older chemotherapies.

Cyclin-dependent kinase 2 (CDK2) is one of the serine/threonine protein kinases. It plays a pivotal role in regulating the cell cycle transition from G1 to S phase, and thus in controlling cell proliferation. Hence, CDK2 inhibitors are potentially effective anti-cancer agents. Although a number of CDK2 inhibitors have been described in the literature [1] and some have entered clinical trial phases, e.g. flavopiridol [2], roscovitine [3] and olomoucine [4], none of them has been approved for clinical use due to various reasons such as toxicity and multi-target specificity. Furthermore, none of the reported CDK2 inhibitors are for the treatment of HCC.

In this study, we used our free and open-source protein-ligand docking software idock [5, 6] to screen FDA-approved small molecule drugs against CDK2, thus avoiding the toxicity problem. We adopted the *in silico* approach of structure-based virtual screening and ensemble docking to repurpose approved drugs for the treatment of cancers that involve CDK2 regulation, with a major focus on human hepatocellular carcinoma (HCC). We tested nine computationally favoured compounds *in vitro* in HCC cell lines HepG2 and Huh7, and successfully identified the anti-psychotic drug fluspirilene as a potential CDK2 inhibitor. We then performed *in vivo* experiments in nude mice xenografted with Huh7 cells, and showed that fluspirilene exhibited strong anti-tumor activity comparable to that of the leading cancer drug 5-fluorouracil, further establishing fluspirilene as a candidate anti-cancer drug. We also showed that the cocktail treatment with both fluspirilene and 5-fluorouracil could produce synergistic therapeutic effect. Finally, we analyzed the predicted binding conformation of fluspirilene and revealed the critical intermolecular interactions that possibly govern fluspirilene binding to CDK2.

## Methods and Materials

### Ethics statement

This study was approved by the laboratory animal ethics committee of Kunming Medical University.

### Ensemble docking and compound selection

There are as many as 346 solved X-ray crystallographic structures of CDK2 from the PDB (Protein Data Bank) [7, 8] with a UniProt ID of P24941 (S1 Table). Among them, we collected 44 crystal structures of CDK2 in complex with a bound ligand (Table 1; S1 Fig). These 44 structures were selected because they do not contain metal ions, which may influence the docking accuracy of idock [6], and also because they contain a bound ligand, whose coordinate helps to define a search space easily. A previously written script [6] was re-used to automatically define the docking search space by finding the smallest cubic box that covers the entire co-crystallized ligand and subsequently extending the box by 10Å in all the three dimensions. The 44 CDK2 structures were manually extracted from their corresponding complexes with the co-crystallized ligands and waters removed, and then converted from PDB format to PDBQT format using the prepare_receptor4.py script of AutoDockTools [9].

**Table 1:** The 44 CDK2 holo structures used for ensemble docking.

PDB ID	Resolution (Å)
1GZ8	1.30
1JVP	1.53
1H00	1.60
1OIT	1.60
1URW	1.60
1H01	1.79
1H08	1.80
1E1X	1.85
1H07	1.85
1H0V	1.90
1OIR	1.91
1E1V	1.95
1PXI	1.95
1JSV	1.96
1PXO	1.96
1AQ1	2.00
1GII	2.00
1KE6	2.00
1KE7	2.00
1KE8	2.00
1KE9	2.00
1PYE	2.00
1R78	2.00
1CKP	2.05
1DM2	2.10
1H0W	2.10
1DI8	2.20
1FVT	2.20
1GIJ	2.20
1KE5	2.20
1W0X	2.20
1VYZ	2.21
1PXJ	2.30
1PXP	2.30
1OIQ	2.31
1V1K	2.31
1P2A	2.50
1PXL	2.50
1PXN	2.50
1PF8	2.51
1PXM	2.53
1G5S	2.61
1GIH	2.80
1PXK	2.80

The final score used to rank compounds was purposely designed to be the average score across these 44 structures of CDK2 so as to account for structural variability.

To test the redocking accuracy of idock, the co-crystallized ligands were also manually extracted from their corresponding complexes and converted from PDB format to PDBQT format using the prepare_ligand4.py script of AutoDockTools [9]. These ligands were then docked back to their corresponding CDK2 structure, and their root mean square deviation (RMSD) from the co-crystallized conformation was calculated.

The structures of FDA-approved drugs were obtained from the dbap and fda catalogs of the ZINC database [10, 11], where the dbap catalog comprises approved drugs collected from the DrugBank database [12] and the fda catalog comprises approved drugs collected via the DSSTox (Distributed Structure-Searchable Toxicity) project. The dbap catalog of version 2014-03-19 with 1,738 compounds and the fda catalog of version 2012-07-25 with 3,176 compounds were downloaded. Among these 4,914 compounds, 4,311 were unique in terms of ZINC ID. These 4,914 compounds in Mol2 format were then converted to PDBQT format using the prepare_ligand4.py script of AutoDockTools [9].

Our free and open-source docking software idock v2.1.2 [6] was then executed to predict the binding conformations and the binding affinities of the 4,914 compounds when docked against the 44 CDK2 structures using an ensemble docking strategy [13–15]. For each protein structure, free energy grid maps with a fine granularity of 0.08 Å were constructed in parallel, and for each compound, 256 Monte Carlo conformational optimization tasks were run in parallel across multiple CPU cores.

After docking, idock outputted a maximum number of nine predicted conformations for each input compound. The docked conformation with the best idock score was selected because it was previously shown to be the most likely one closest to the crystal conformation with a redocking success rate of more than 50% on three different benchmarks [6]. The 4,914 compounds were sorted in the ascending order of their predicted binding free energy averaged across the 44 CDK2 structures, and the top-scoring ones were visually examined using iview [16] in the context of CDK2 using the X-ray crystal structure of the highest resolution, i.e. PDB ID 1GZ8 in this case (Table 1). Next, commercially available compounds were queried and purchased via the Chemical Book website http://www.chemicalbook.com/ and subsequently validated *in vitro* and *in vivo*.

### Chemicals, antibodies, cell lines and cell culture

The selected chemicals and the leading cancer drug 5-fluorouracil were purchased from Sigma-Aldrich, USA. Anti-cyclin D, B1, E, CDK2, Rb, pho-CDK2 (Thr160), pho-Rb (Ser795) and GAPDH were obtained from Cell Signaling Technology, Inc., Danvers, Massachusetts, USA.

Hepatoma cell lines HepG2 and Huh7 were obtained from the American Type Culture Collection, Manassas, Virginia, USA. These cell lines were cultured in RPMI 1640 medium containing 10% fetal bovine serum (FBS) (Invitrogen, Rockville, Maryland, USA) at 37°C in 5% CO_2_ and 95% humidified air.

Cells were plated in 96-, 24-, or 6-well plates with 0.125% FBS medium for 24 hours and then treated with 10% FBS medium containing the testing compounds at various concentrations of 1, 3, 10, 30 *μ*M, and incubated for 24, 48, or 72 hours.

### MTT assay

Cells were plated at an initial density of 9 × 10^3^ cells/well in 96 well plates and incubated with 0.5mg/ml 3-(4,5-methylthiazol-2-yl)-2,5-diphenyl-tetrazolium bromide for 4 hours. The medium was then discarded and 200 *μ*l of formazan in dimethylsulphoxide (DMSO) was added. The absorbance was measured at 570 nm according to the standard protocol. The IC_50_ values were calculated by GraphPad Prism 5.

### Cell cycle analysis

HepG2 or Huh7 cells (4 × 10^4^) were seeded in 24-well plates in RPMI 1640 medium containing 0.125% FBS, and cultured for 24 hours. The cells were incubated in medium containing 10% FBS and various doses of fluspirilene (1, 3, 10, 30 *μ*M) for 12, 24, 36 hours at 37°C, then fixed in ice-cold 70% ethanol and stained with a Coulter DNA-Prep Reagents kit (Beckman Coulter, Fullerton, California, USA). Cellular DNA content of 1 × 10^4^ cells from each sample was determined with the use of an EPICS ALTRA flow cytometer (Beckman Coulter). Cell cycle phase distribution was analyzed with the ModFit LT 2.0 software (Verity Software House, Topsham, Maine, USA). All results were obtained from two separate experiments, each of which was done in triplicate.

### Cell apoptosis analysis

HepG2 and Huh7 cells were plated at 24-well plates with 0.125% FBS medium for 24 hours and then with 10% FBS medium containing fluspirilene at concentrations 3, 10 and 30 *μ*M. Following the manufacturer’s instructions to quantify the apoptotic cells, the occurrence of apoptosis was determined by staining cells with both annexin V and propidium iodide (PI) (Life Technologies). Briefly, cells were trypsinized with 0.25% trypsin in the absence of ethylenediamine tetraacetic acid (EDTA). The cells were washed with PBS twice and resuspended in 500 *μ*l of binding buffer at a concentration of 2 × 10^5^ − 10^6^ cells/ml. Two microliters of annexin V-EGFP and 5 *μ*l of PI were added to the suspension followed by 5 to 15 minutes of incubation in the dark. The cells were then analyzed using flow cytometry (CyFlow Space/Partec, Germany).

### Western blotting

HepG2 and Huh7 cells were plated at 6-well plates with 0.125% FBS medium for 24 hours and then with 10% FBS medium containing fluspirilene at concentration 3, 10, 30 *μ*M. Cells were harvested after 6 hours of incubation. Cells were lysed with RIPA buffer containing 1 mM PMSF and protease inhibitor cocktail at 4°C for 30 minutes. After centrifugation at 13,000 rpm for 15 minutes, the supernatants were recovered and the protein concentration was measured by BCA Protein Assay Kit (Thermo). Equal amounts of cell lysates were resolved in 10% SDS-PAGE and transferred onto nitrocellulose membranes (Sigma). After blocking, the membranes were incubated sequentially with the appropriate diluted primary and secondary antibodies. Proteins were detected by the enhanced chemiluminescence detection system (Amersham, Piscataway, New Jersey, USA). To ensure equal loading of the samples, the membranes were re-probed with an anti-GAPDH antibody (Cell Signalling Technologies).

### Fluspirilene treatment *in vivo*

Female BALB/C nude mice, 4 to 5 weeks old from Vital River Laboratory Technology Co. Ltd, Peking, China, were kept under specific pathogen-free conditions. For the xenografted tumor growth assay, 1 × 10^6^/0.2ml PBS Huh7 cells were injected subcutaneously into the right flank of the mice. Tumor size was measured every day. Three weeks after inoculation when the tumors grew to a volume of 80 to 100 m^3^, the mice were randomly divided into groups of 5 mice per group, and fed by oral gavage with 0.5% CMC-NaCl containing fluspirilene (15mg/kg) and by intraperitoneal injection of 5-fluorouracil (10mg/kg) daily for 21 days. Carcinoma volumes were measured every 3 to 4 days after tumor appearance. Tumor volume was calculated by the formula *V* = *ab* ^2^/2, where *a* is the longest axis and *b* is shortest axis. The mice were then sacrificed by cervical dislocation.

### Statistical analysis

The results were obtained from at least three different experiments and expressed as mean ± SD. Statistical analysis was performed by Student’s t test and differences were considered to be statistically significant if p < 0.05. Statistically significant results are marked with the * symbol in the figures.

## Results

### Redocking results

For the 44 CDK2 complexes, the co-crystallized ligands were docked against their corresponding CDK2 structure to see if idock could predict binding poses as conformationally close to the co-crystallized pose as possible. Such redocking accuracy was quantified by the root mean square deviation (RMSD) of the predicted poses from the co-crystallized pose. For each of the 44 complexes, idock predicted nine poses, hence nine RMSD values. The RMSD values of the nine poses in the 44 redocking trials are provided in S2 Table.

**Fig 1:**
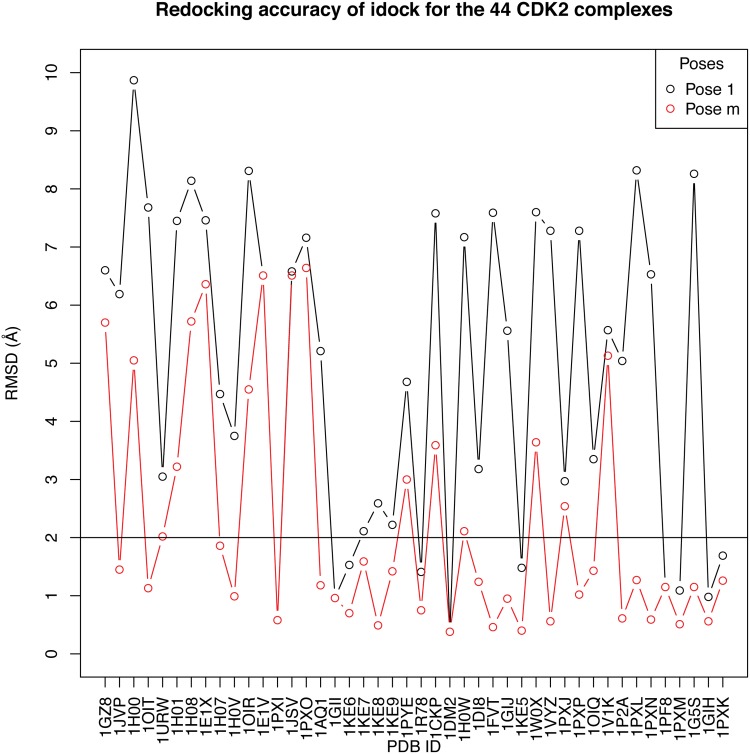
Redocking accuracy of idock for the 44 CDK2 complexes. The RMSD distributions of pose 1 and pose m are shown in black and red, respectively. 10 and 28 points are below the baseline of RMSD = 2Å for pose 1 and pose m, respectively.

Fig 1 plots the distribution of RMSD values of the first pose (denoted as pose 1), which was predicted to have the lowest free energy among the nine poses, as well as the distribution of RMSD values of the pose that has the minimum RMSD among the nine poses (denoted as pose m). Detailed explanation and the rationale of selection of these two kinds of poses can be found in [6]. Semantically, the RMSD value of pose 1 (denoted as RMSD1) and the RMSD value of pose m (denoted as RMSDm) reflect the redocking accuracy if only the top one and top nine predicted poses are considered, respectively. By definition, RMSDm ≤ RMSD1 is always guaranteed. Out of the 44 redocking trials, 10 trials produced RMSD1 < 2Å and 28 trials produced RMSDm < 2Å. These redocking results reflect to some extent the appropriateness of using idock to dock compounds against CDK2 structures.

### Ensemble docking results and selection of candidate inhibitors

Totally 4,914 FDA-approved drugs were docked and ranked according to their average predicted binding affinity across 44 X-ray crystal structures of CDK2. Their predicted free energy values are provided in S3 Table. The docking prediction results with iview visualization [16] are freely available at http://istar.cse.cuhk.edu.hk/idock/iview/?1GZ8-dbap and http://istar.cse.cuhk.edu.hk/idock/iview/?1GZ8-fda.

**Fig 2:**
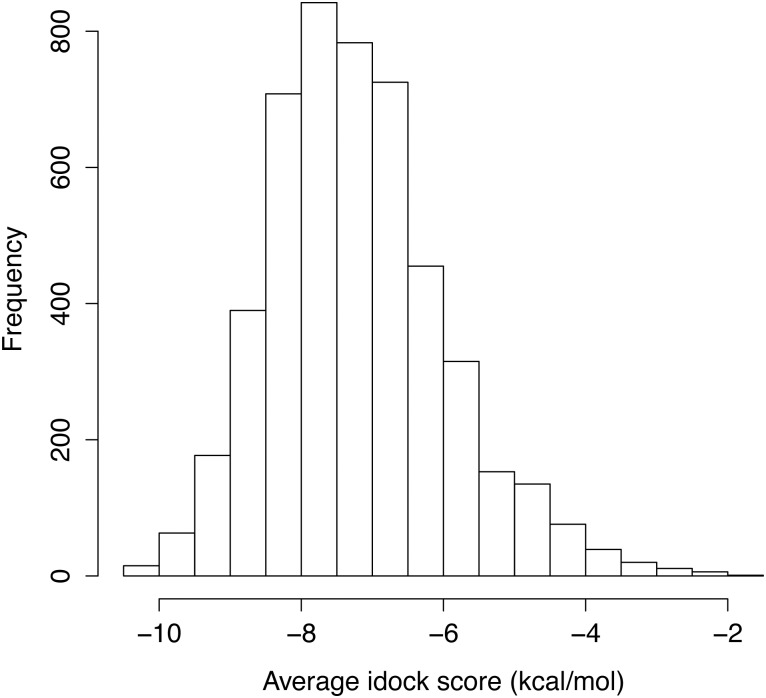
Histogram distribution of the average idock score of the 4,914 compounds. 842 and 783 compounds were in the ranges of [−7.5, −8.0) and [−7.0, −7.5), respectively, constituting the two largest bins. 15 and 63 compounds were in the ranges of [−10.0, −10.5) and [−9.5, −10.0), respectively, constituting the two bins appropriate for selection of candidate drugs.

**Fig 3:**
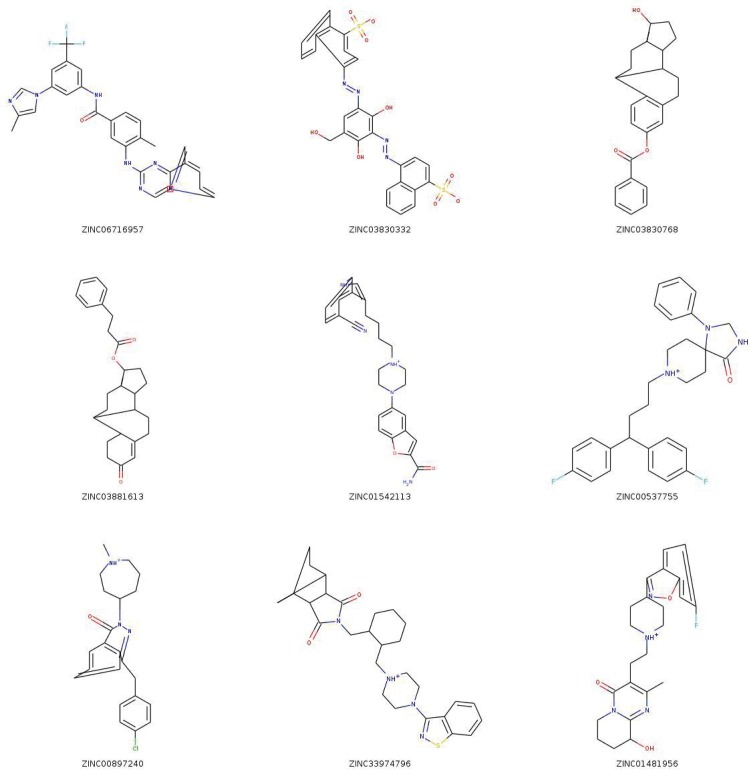
The 2D structures of the nine top-scoring compounds. This figure was created by RDKit (http://rdkit.org/).

Fig 2 plots the distribution of the average idock score of the 4,914 compounds. 15 compounds had predicted free energy of −10 kcal/mol or lower, with the best one having −10.46 kcal/mol. Based on commercial availability, 9 top-scoring compounds (Table 2; Fig 3; S4 Table) were purchased for further investigations.

**Table 2:** The nine top-scoring compounds purchased and tested *in vitro*.

ZINC ID	average score	standard deviation	name
06716957	−10.46	0.52	nilotinib
03830332	−10.43	0.50	LS-194959
03830768	−10.23	0.75	estradiol benzoate
03881613	−10.08	0.65	nandrolone phenylpropionate
01542113	−10.06	0.51	vilazodone
00537755	−10.02	0.66	fluspirilene
00897240	−10.01	0.65	azelastine hydrochloride
33974796	−9.98	0.63	latuda
01481956	−9.95	0.50	paliperidone

The idock score is an estimation of binding free energy in kcal/mol units. A more negative value implies a higher predicted binding affinity.

### Fluspirilene decreased cell viability of hepatocellular carcinoma

**Fig 4:**
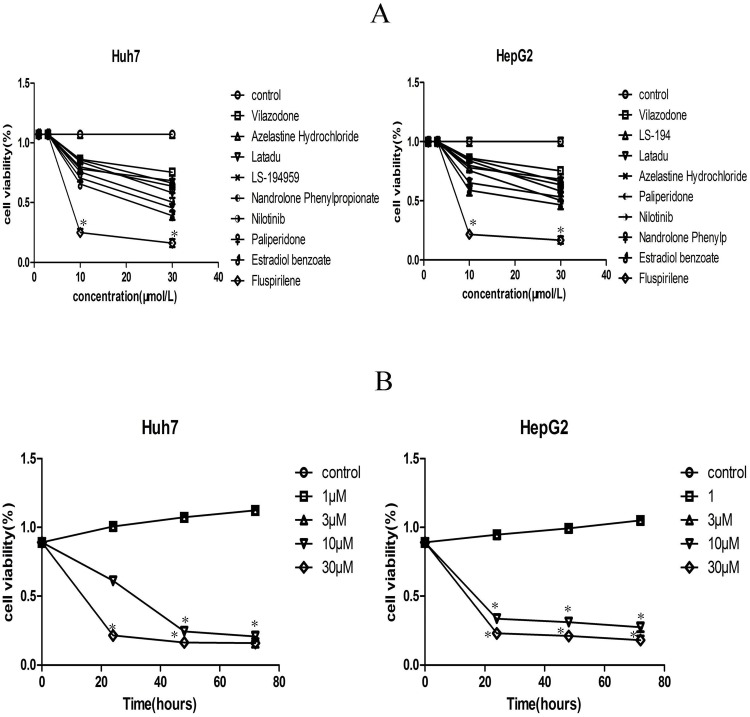
Effect of the nine compounds on the viability of HepG2 and Huh7 hepatocellular carcinoma cells. (A) The nine compounds had discrepant cytotoxicity to HepG2 and Huh7 cell lines at different concentrationas, with fluspirilene exhibiting the highest cytotoxicity compared to the control (p < 0.05). (B) Fluspirilene exhibited dose- and time-dependent inhibition on cell viability in HepG2 and Huh7 cell lines compared to the control (p < 0.05).

We first evaluated the anti-cancer effect of the nine compounds by MTT assay (Fig 4). All the nine compounds decreased cell viability in HepG2 and Huh7 cells, but had discrepant cytotoxicity at different concentrations. Among them, fluspirilene had the lowest IC_50_, i.e. 4.017 *μ*M for HepG2 and 3.468 *μ*M for Huh7. Fluspirilene exhibited the highest cytotoxicity compared to the control with statistical significance (p < 0.05). Such inhibition effect was dose- and time-dependent. Marked inhibition was observed at 10 *μ*M and 30 *μ*M, but no significant effect was observed at concentrations below 3 *μ*M.

### Fluspirilene treatment arrested cell cycle in G1 phase

**Fig 5:**
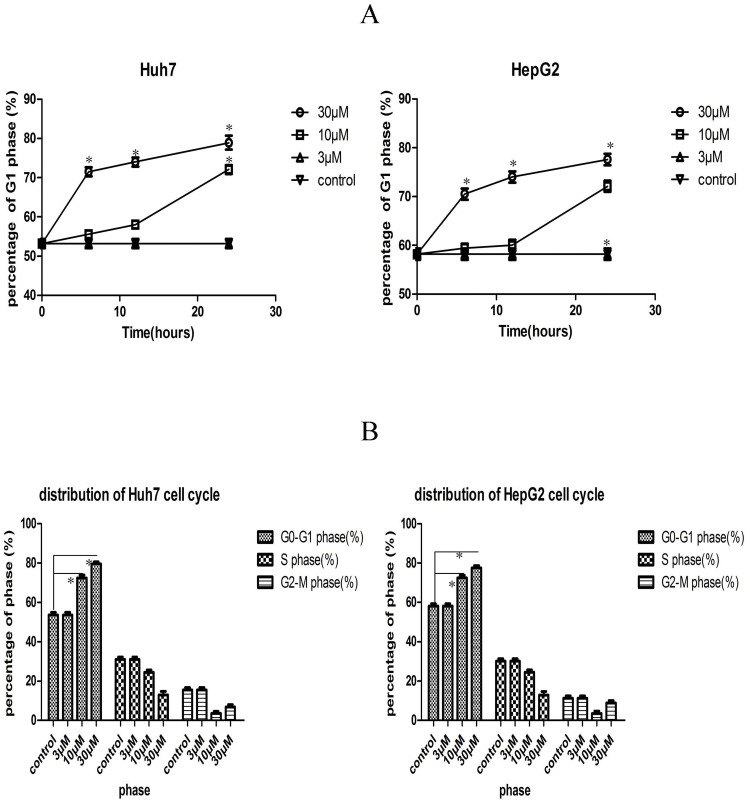
Dose- and time-dependent effect of fluspirilene treatment on cell cycle profile of G0-G1, S, and G2-M phases. (A) Fluspirilene treatment dose- and time-dependently increased the percentage of cells in G1 phase. At 30 *μ*M or 10 *μ*M concentration, fluspirilene treatment continuously increased the percentage of G1 phase for 24 hours. (B) Cell cycle distribution at 24 hours after fluspirilene treatment. The increase of the G1 phase was accompanied by the simultaneous decrease of S phase.

To understand if fluspirilene inhibited CDK2 activities in hepatocellular carcinoma cells, we analyzed the effect of fluspirilene treatment with concentrations of 3, 10, 30 *μ*M for 6, 12, 24 hours on cell cycle profile in HepG2 and Huh7 cells by flow cytometry (Fig 5). Fluspirilene treatment significantly increased the percentage of cells in G1 phase compared to the control (p < 0.05) in a dose- and time-dependent manner. At 30 *μ*M or 10 *μ*M concentration, fluspirilene treatment continuously increased the percentage of G1 phase for 24 hours. After 24 hours of fluspirilene treatment, the increase of G1 phase was accompanied by the simultaneous decrease of S phase. The sub-G1 percentages are provided in S5 Table. The representative histograms of DNA content are provided in S5 Fig.

### Fluspirilene treatment induced cell apoptosis

**Fig 6:**
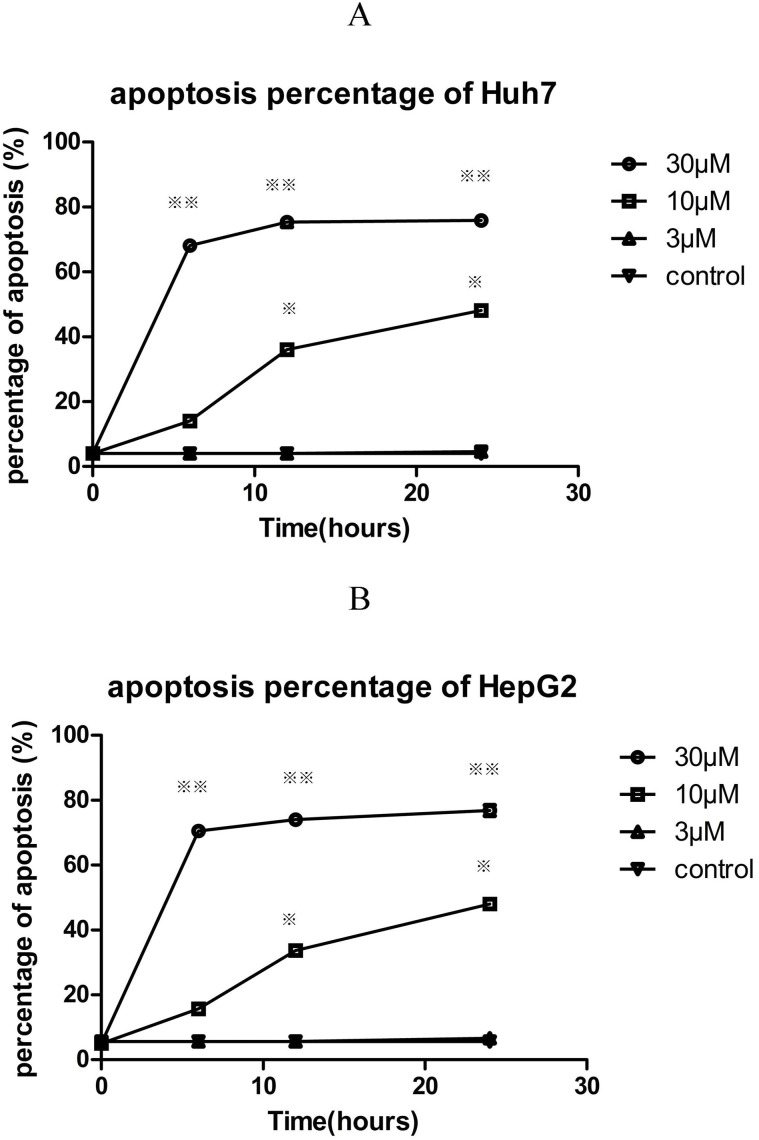
Effect of fluspirilene on the apoptosis of HepG2 and Huh7 hepatocellular carcinoma cells. Fluspirilene treatment at 30 *μ*M or 10 *μ*M concentration significantly increased the percentage of apoptosis in Huh7 and HepG2 cell lines compared to the control in a dose-and time-dependent manner (p < 0.05).

A drug that can only induce cell cycle arrest is not good enough because the tumors will easy re-grow after withdrawing the drug. Therefore we also investigated whether fluspirilene could induce cell apoptosis (Fig 6). Fluspirilene treatment at 30 *μ*M or 10 *μ*M concentration significantly increased the percentage of apoptosis in Huh7 and HepG2 cell lines compared to the control in a dose-and time-dependent manner (p < 0.05). The scatter plots of PI against annexin V are provided in S6 Fig.

### Fluspirilene treatment decreased the expressions of CDK2, Rb, cyclin E, pho-CDK2 and pho-Rb, but not cyclin D1 and cyclin B1

**Fig 7:**
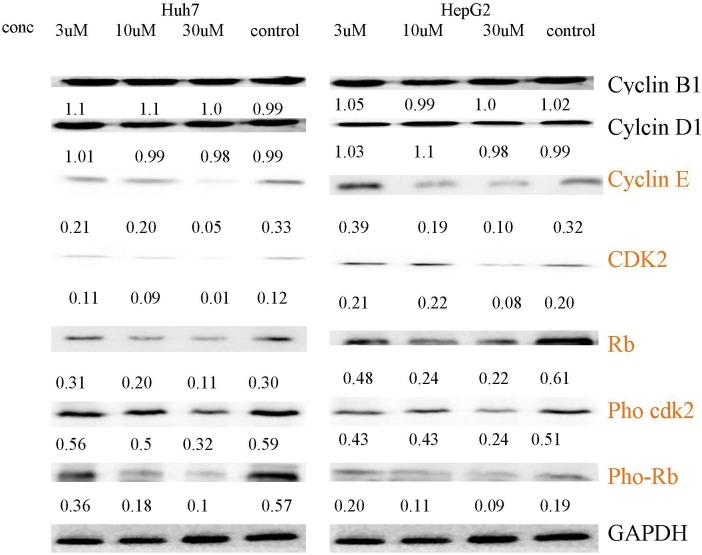
Effect of fluspirilene treatment on the expressions of important proteins involved in G1-to-S transition. Fluspirilene treatment decreased the expressions of CDK2, Rb, cyclin E, pho-CDK2 and pho-Rb, but not cyclin D1 and cyclin B1.

We investigated the effect of fluspirilene on the expressions of critical proteins involved in G1-to-S transition in HepG2 and Huh7 cells by western blotting (Fig 7). Fluspirilene treatment reduced the expressions of CDK2, Rb, pho-CDK2, pho-Rb and cyclin E. In contrast, the expression levels of cyclin D1 and cyclin B1 remained unchanged. These results are consistent with what are expected from a CDK2 inhibitor.

It is also observed that fluspirilene treatment majorly decreased the expression rather than phosphorylation of CDK2. We propose that such differences are reasonable in the sense that after binding, CDK2-fluspirilene complexes may directly undergo degradation, therefore resulting in higher reduction of CDK2 expression than phosphorylation. In addition, fluspirilene may bind to CDK2 with a high affinity, but after CDK2 is phosphorylated, the binding of fluspirilene may be impaired and the binding affinity could thus be weakened.

### Daily oral fluspirilene treatment reduced tumor growth *in vivo*

**Fig 8:**
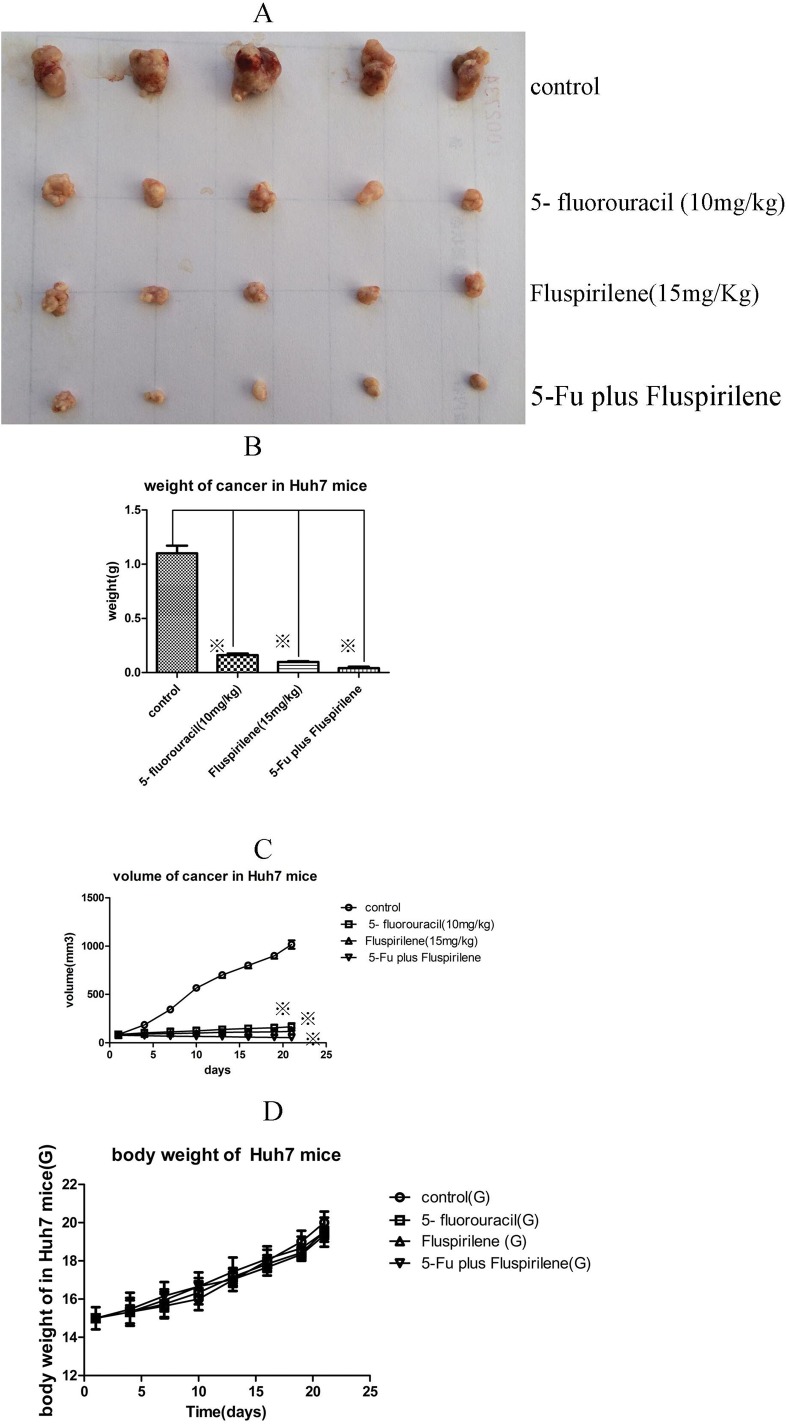
Effects of oral fluspirilene treatment and intraperitoneal injection of 5-fluorouracil on tumor growth *in vivo* in nude mice xenografted with Huh7 cells. The anti-tumor activity of oral fluspirilene (15 mg/kg) was comparable to that of 5-fluorouracil (10 mg/kg). Furthermore, their cocktail therapy exhibited synergistic therapeutic effect.

We evaluated the effect of fluspirilene on the growth of hepatocellular carcinoma *in vivo* in BALB/C nude mice subcutaneously injected with Huh7 cells (Fig 8). On day 21 after treatment, fluspirilene (15 mg/kg) resulted in significant reduction of tumor weight and volume compared to the control (p < 0.05), while making no significant change to the body weight. The anti-tumor activity of oral fluspirilene (15 mg/kg) was comparable to that of 5-fluorouracil (10 mg/kg). Importantly, their combined therapy exhibited the highest therapeutic effect. These results suggested for the first time that fluspirilene is a potential CDK2 inhibitor and a candidate anti-cancer drug for the treatment of human hepatocellular carcinoma.

### Structural analysis of the predicted binding conformation of fluspirilene

**Fig 9:**
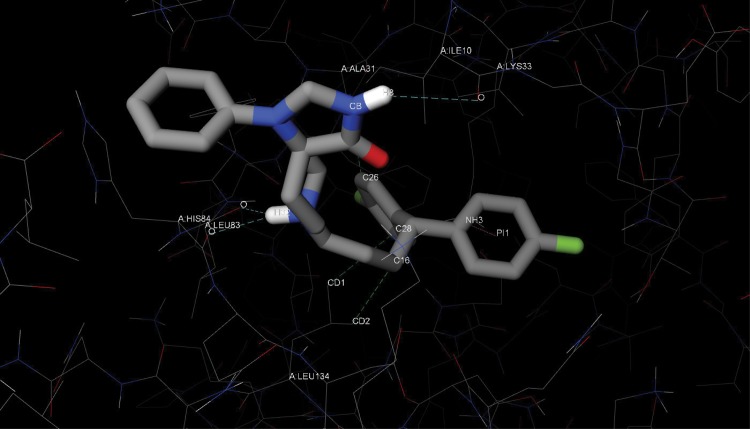
The predicted conformation of fluspirilene in complex with CDK2. CDK2 residues are rendered as lines colored by atom type. Fluspirilene is rendered as sticks colored by atom type. The interacting atoms and residues are labeled. The cyan, green and pink dashed lines represent hydrogen bonds, hydrophobic contacts and *π* interactions, respectively. This figure was created by iview [16].

Fig 9 plots the predicted conformation of fluspirilene in complex with CDK2 (PDB ID: 1GZ8) using iview [16]. Fluspirilene was predicted to bind inside the ATP-binding pocket of CDK2 and interact with CDK2 mainly through hydrogen bonds, hydrophobic contacts and cation-*π* interactions. Putatively, the H8 atom forms a hydrogen bond with the backbone oxygen of Ile10, and the H32 atom forms another two hydrogen bonds with the backbone oxygen of Leu83 and His84. The side chains of Ile10, Ala31 and Leu134 establish a hydrophobic tunnel for the hydrophobic tail of fluspirilene to bury inside. One of the two aromatic rings located at the tail of fluspirilene forms a cation-*π* interaction with the positively charged side chain of Lys33. All these putative hydrogen bonds, hydrophobic contacts and cation-*π* interactions are spread over the head, middle and tail fragments of fluspirilene, thereby firmly holding fluspirilene at its predicted position and orientation. In comparison, the known CDK2 inhibitor 2-Amino-6-(3’-methyl-2’-oxo)butoxypurine (codenamed MBP for short), which is an O(6)-substituted guanine derivative, is known to establish four hydrogen bonds with Lys33, Glu81 and Leu83 (S2 Fig).

We further inspected the predicted conformation of fluspirilene in the presence of CDK2 molecular surface (S3 Fig). Compared with the guanine derivative MBP (S4 Fig), fluspirilene occupies a much larger portion of the binding pocket, and its shape properly matches that of the binding cavity. Based on this structural analysis together with our *in vitro* and *in vivo* experiments, we infer that fluspirilene may physically bind to CDK2.

## Discussion

Cell cycle progress is sequentially and strictly processed through the interactions of CDKs and cyclins [17]. Different cyclin-CDK complexes are activated in different phases of the cell cycle. When the cell cycle goes through G1 to S phase, the cyclin D1-CDK4/6 and cyclin E-CDK2 complexes are ordinally activated and the retinoblastoma protein (pRB) is hyper-phosphorylated on serine and threonine residues [18]. The hyper-phosphorylated pRB promotes the release of E2F transcription factors, which in turn facilitate the transcription of numerous genes required for G1 to S transition and S phase progression [19]. From the medicinal perspective, CDK2 has long been a classical and important target for cancer therapy.

Though a number of CDK2 inhibitors have entered clinical trial phases, none has been officially approved for clinical use, probably because of their toxicity and multi-target specificity. Given the obstacle that developing a new drug *de novo* is a laborious and costly endeavor, repurposing toxicity-free old drugs for new uses is a favorable strategy.

The powerful synergy of *in silico* methods in drug repurposing by structure-based virtual screening (SBVS) was highlighted in several recent publications [20]. To name a few successful repurposing cases by SBVS, [21] rediscovered 2,4-Dichlorophenoxy acetic acid, a well-known plant auxin, as a new anti-inflammatory agent through *in silico* molecular modeling and docking studies along with drug formulation and *in vivo* anti-inflammatory inspection; [22] attempted to repurpose FDA-approved drugs by an integrated SBVS approach and reported the discovery of piperacillin **1** as an inhibitor of NEDD8-activating enzyme (NAE) in cell-free and cell-based systems with high selectivity.

In addition to SBVS, ligand-based virtual screening (LBVS) also finds its successful applications in repurposing. [23] used Ultrafast Shape Recognition (USR) [24] to search for compounds with similar shape to a previously reported inhibitor of protein arginine deiminase type 4 (PAD4), a new therapeutic target for the treatment of rheumatoid arthritis, and identified a novel compound that has a strikingly different structure from the template inhibitor yet showed significant inhibition on the enzymatic activity of PAD4.

Encouraged by these successful stories, in this study we adopted the repurposing strategy, and utilized the computational methodology of SBVS by protein-ligand docking to shortlist candidates from FDA-approved small molecule drugs. Specifically, we used our fast docking program idock [5, 6] in combination with our convenient visualizer iview [16] for the task of rediscovering existing drugs as CDK2 inhibitors. idock is an exciting development not only because it has been vigorously shown [6] to outperform the state-of-the-art docking software AutoDock Vina [25] in terms of docking speed by at least 8.69 times and at most 37.51 times while maintaining comparable redocking success rates, but also because it is free and open source under a permissive license. The latter guarantees that users from both industry and academia can freely utilize idock in their own projects that require protein-ligand docking.

To facilitate the use of idock, its input arguments and output results were purposely designed to be similar to those of AutoDock Vina, therefore existing users can easily transit to idock and benefit from considerable speedup in SBVS performance. Moreover, to promote prospective SBVS by idock, a web server called istar [6] was developed and made freely available at http://istar.cse.cuhk.edu.hk/idock, where there are as many as over 23 million purchasable small molecule compounds ready for docking against any protein supplied by the user. As of 1 May 2015, there have been more than 1000 docking job submissions by users from 98 countries. Both idock [5] and istar [6] would hopefully supplement the efforts of medicinal chemists in drug discovery research. Besides for research purpose, some universities also use our idock for teaching purpose in their bioinformatics courses.

Regarding the structural data in use, so far there are as many as 346 solved X-ray crystal structures of CDK2 with a UniProt ID of P24941 (S1 Table). To account for their structural variability and to mine knowledge from multiple structures of CDK2, we selected 44 holo structures of CDK2 in a bound state with a ligand in complex to carry out ensemble docking. The final score used to prioritize compounds was purposely designed to be the average score of that compound when docked to the 44 selected structures of CDK2 with their co-crystallized ligand removed manually before docking. In this way the top-scoring compounds would guarantee a consistent binding strength on average. Moreover, the standard deviation of scores across the 44 CDK2 structures was no greater than 0.8 kcal/mol for the top-scoring compounds (Table 2; S3 Table), further indicating that the average score is a reliable metric. In the aspect of data source of approved drugs, although we chose the dbap and fda catalogs of the ZINC database [10, 11], it is also possible to use some other freely accessible drug databases such as NCGC [26], DrugBank [12], KEGG DRUG [27] and e-Drug 3D [28].

After ensemble docking experiments with idock [5, 6] followed by careful visual inspections with iview [16], we purchased nine top-ranking compounds for subsequent wet experiments. Among them, fluspirilene was selected for further investigations because its IC_50_ was less than 10 *μ*mol/L as determined by MTT assay. Fluspirilene is currently used for the therapy of chronic schizophrenia. It was recently identified as a potential p53-MDM2 inhibitor and its anti-cancer effect *in vitro* was reported [29], but was never investigated *in vitro* in HCC cell lines, nor *in vivo* in nude mice, and its role as a potential CDK2 inhibitor remained unknown until now.

In this study, we reported for the first time that fluspirilene is a potential CDK2 inhibitor, and demonstrated for the first time that oral administration of fluspirilene (15 mg/kg) exhibited significant and strong anti-cancer efficacy comparable to the leading cancer drug 5-fluorouracil (10 mg/kg) *in vivo* in nude mice xenografted with hepatoma Huh7 cells. Most importantly, the combination of effective dose of fluspirilene and 5-fluorouracil produced even higher therapeutic effect, indicating that fluspirilene may work through a different mechanism than 5-fluorouracil, which further indicates that fluspirilene could be combined with other chemotherapy drugs to achieve synergistic therapeutic effect. Our finding is particularly inspiring in that fluspirilene could be the first CDK2 inhibitor used for HCC therapy.

No obvious toxicity was previously reported by intraperitoneal injection of fluspirilene (8 mg/kg) in male wistar rats [30]. In this study, we did not observe significant change in body weight by oral administration of fluspirilene (15 mg/kg) for 21 days (Fig 8), suggesting that oral administration or intraperitoneal injection of fluspirilene is relatively safe. The inhibitory rate of 5-fluorouracil (10 mg/kg) on day 2 in female BALB/C *in situ* hepatocellular carcinoma models with HCM-Y89 cell by intravenous injection was 48.14% at 20 day of post-treatment [31]. In this study, we found that the inhibitory rate of 5-fluorouracil (10 mg/kg) on day 21 in female BALB/C nude mice xenografted models with Huh7 cells by intraperitoneal injection was 85.4%, without showing significant body weight change, suggesting that intraperitoneal administration of 5-fluorouracil is relatively safe and effective.

Last but not the least, although we tested fluspirilene *in vitro* and *in vivo* in HCC cells only, we believe fluspirilene may also exhibit anti-cancer effect in other types of cancers.

## Conclusions

This study presents a successful prospective application of idock [5, 6] in identifying CDK2 inhibitors from FDA-approved small molecule drugs using a repurposing strategy and an ensemble docking methodology. We showed that fluspirilene, currently used for the therapy of chronic schizophrenia, exhibited anti-cancer effect in human hepatoma HepG2 and Huh7 cells. We demonstrated for the first time that oral fluspirilene treatment significantly inhibited tumor growth. Most importantly, the combined therapy of fluspirilene and the leading cancer drug 5-fluorouracil produced higher therapeutic effect. These results suggested for the first time that fluspirilene is a potential CDK2 inhibitor and a candidate anti-cancer drug for the treatment of human hepatocellular carcinoma (HCC). Considering the fact that fluspirilene has a long history of safe human use, our discovery of fluspirilene as a potential anti-HCC drug may present an immediately-applicable clinical therapy. The potential application of fluspirilene combined with other chemotherapy drugs for the treatment of hepatoma neoplasms and other cancers warrants further studies.

## Supporting Information

S1 Table The 346 solved X-ray crystal structures of CDK2 with a UniProt ID of P24941. The table comprises the PDB ID, resolution, chain and positions.
(CSV)

S1 Fig Crystal structure of human CDK2 with ATP (PDB ID: 1HCK). The molecular surface of CDK2 is colored by secondary structure, with an opacity of 0.9 to show the underlying secondary structure in cylinder & plate representation. ATP is rendered in stick representation colored by atom type. Waters are shown as red dots and metal ions are shown as green dots. This figure was created by iview [16].
(PNG)

S2 Table Redocking results for the 44 CDK2 complexes. The table comprises the PDB ID, the pose number, the idock score and RMSD.
(CSV)

S3 Table Ensemble docking results of the 4,914 compounds. The table comprises the ZINC ID, the catalog, the average score, standard deviation and individual scores for the 44 selected CDK2 structures, and the molecular properties.
(CSV)

S4 Table Details of the nine top-scoring compounds purchased and tested *in vitro*. The table comprises the ZINC ID, the average score, scientific name, clinical uses and references.
(PDF)

S2 Fig The crystal conformation of MBP in complex with CDK2 (PDB ID: 1GZ8). CDK2 residues are rendered as lines colored by atom type. MBP is rendered as sticks colored by atom type. The interacting atoms and residues are labeled. The cyan dashed lines represent hydrogen bonds. This figure was created by iview [16].
(PNG)

S3 Fig The predicted conformation of fluspirilene in the presence of CDK2 molecular surface. CDK2 is rendered as molecular surface colored by atom type, with an opacity of 0.9 to show the underlying atoms. Fluspirilene is rendered as sticks colored by atom type. This figure was created by iview [16].
(PNG)

S4 Fig The crystal conformation of MBP in the presence of CDK2 molecular surface. CDK2 is rendered as molecular surface colored by atom type, with an opacity of 0.9 to show the underlying atoms. MBP is rendered as sticks colored by atom type. This figure was created by iview [16].
(PNG)

S5 Table Sub-G1 percentages in the cell cycle assay. (PDF)

S5 Fig Representative histograms of DNA content in the cell cycle assay. (JPG)

S6 Fig Scatter plots of PI against annexin V in the cell apoptosis assay. (JPG)
