# Supplementary figures and images for: In Silico Identification and In Vitro and In Vivo Validation of Anti-Psychotic Drug Fluspirilene as a Potential CDK2 Inhibitor and a Candidate Anti-Cancer Drug
Source: PLoS One. 2015 Jul 6;10(7):e0132072. doi: 10.1371/journal.pone.0132072 (PMC4493148; doi:10.1371/journal.pone.0132072)

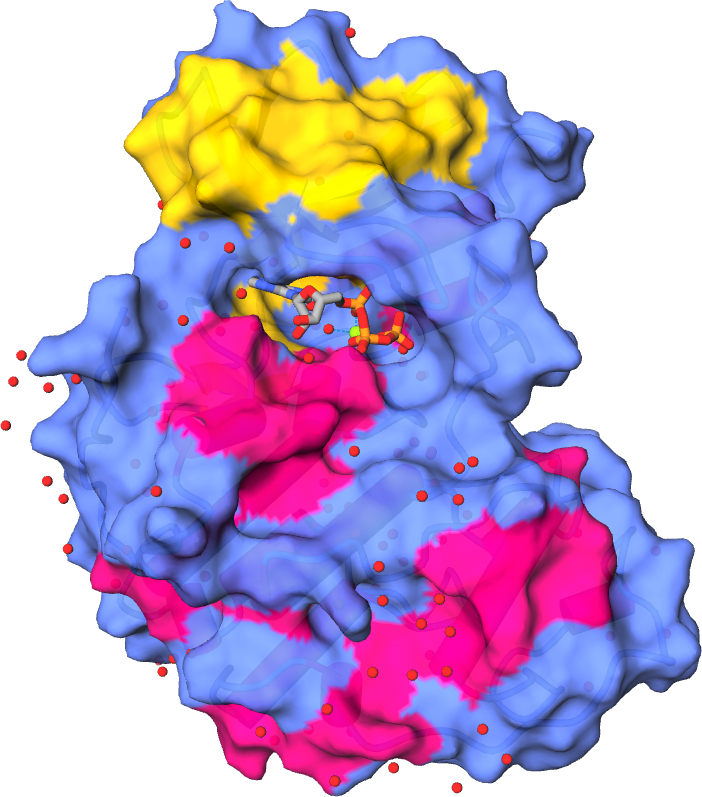

Supplement: S1 Fig — The molecular surface of CDK2 is colored by secondary structure, with an opacity of 0.9 to show the underlying secondary structure in cylinder & plate representation. ATP is rendered in stick representation colored by atom type. Waters are shown as red dots and metal ions are shown as green dots. This figure was created by iview [16]. (PNG) [file pone.0132072.s002.png]

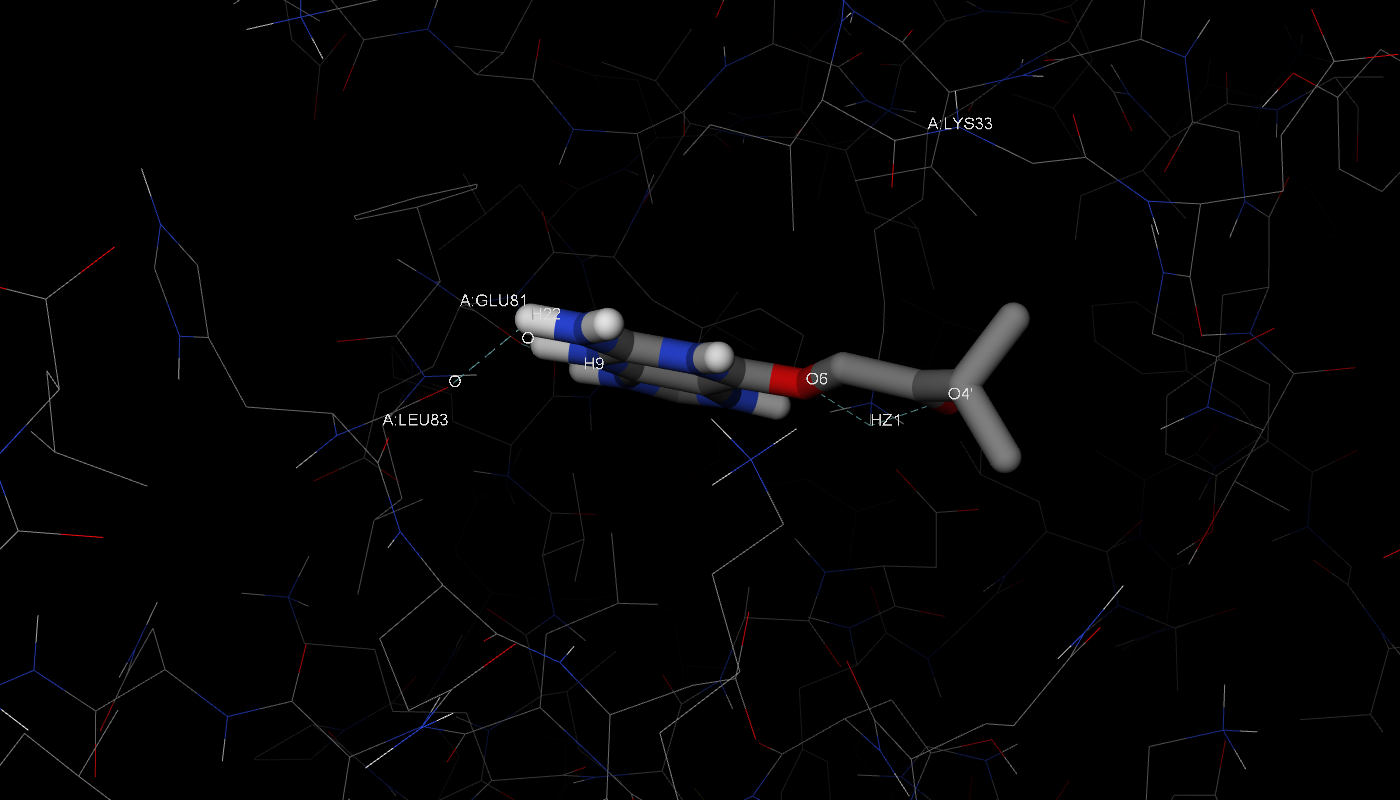

Supplement: S2 Fig — CDK2 residues are rendered as lines colored by atom type. MBP is rendered as sticks colored by atom type. The interacting atoms and residues are labeled. The cyan dashed lines represent hydrogen bonds. This figure was created by iview [16]. (PNG) [file pone.0132072.s006.png]

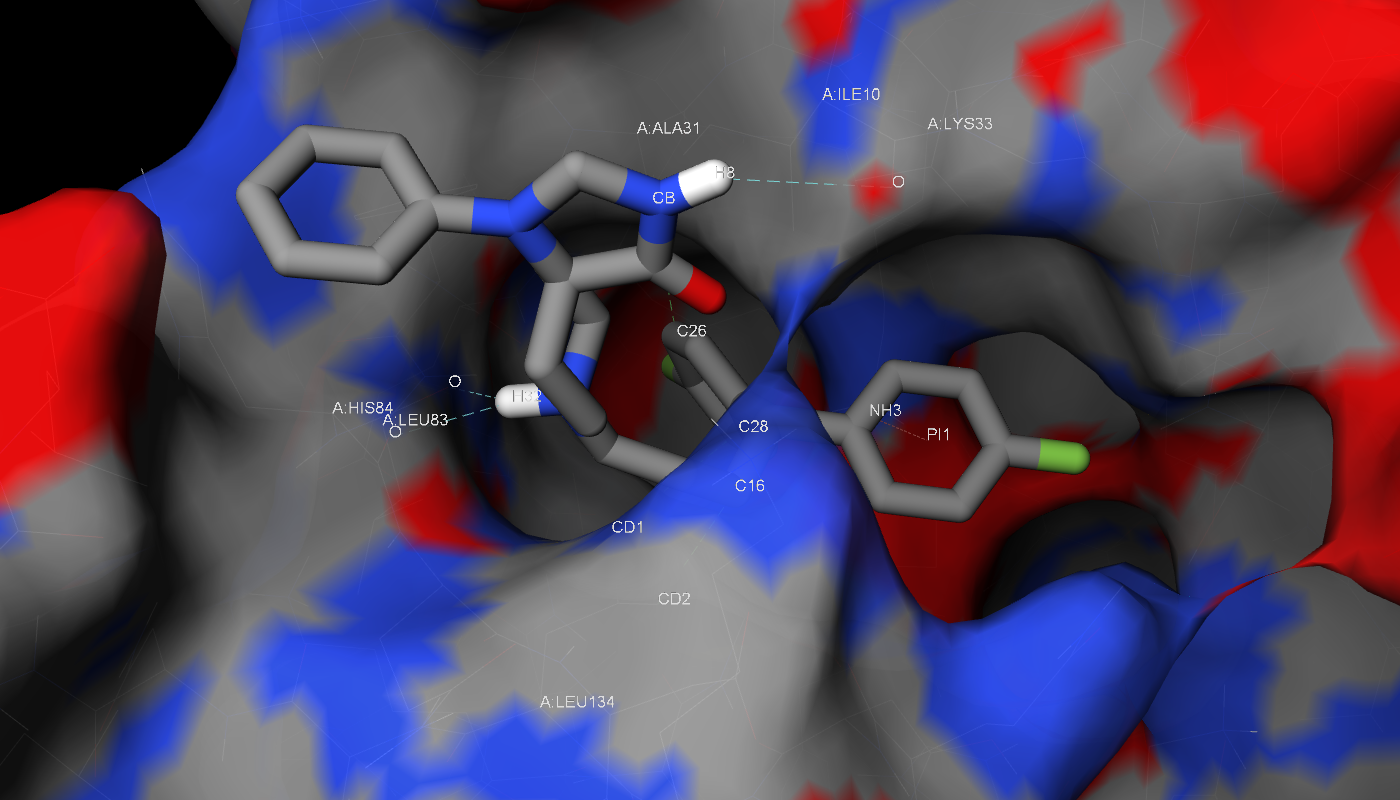

Supplement: S3 Fig — CDK2 is rendered as molecular surface colored by atom type, with an opacity of 0.9 to show the underlying atoms. Fluspirilene is rendered as sticks colored by atom type. This figure was created by iview [16]. (PNG) [file pone.0132072.s007.png]

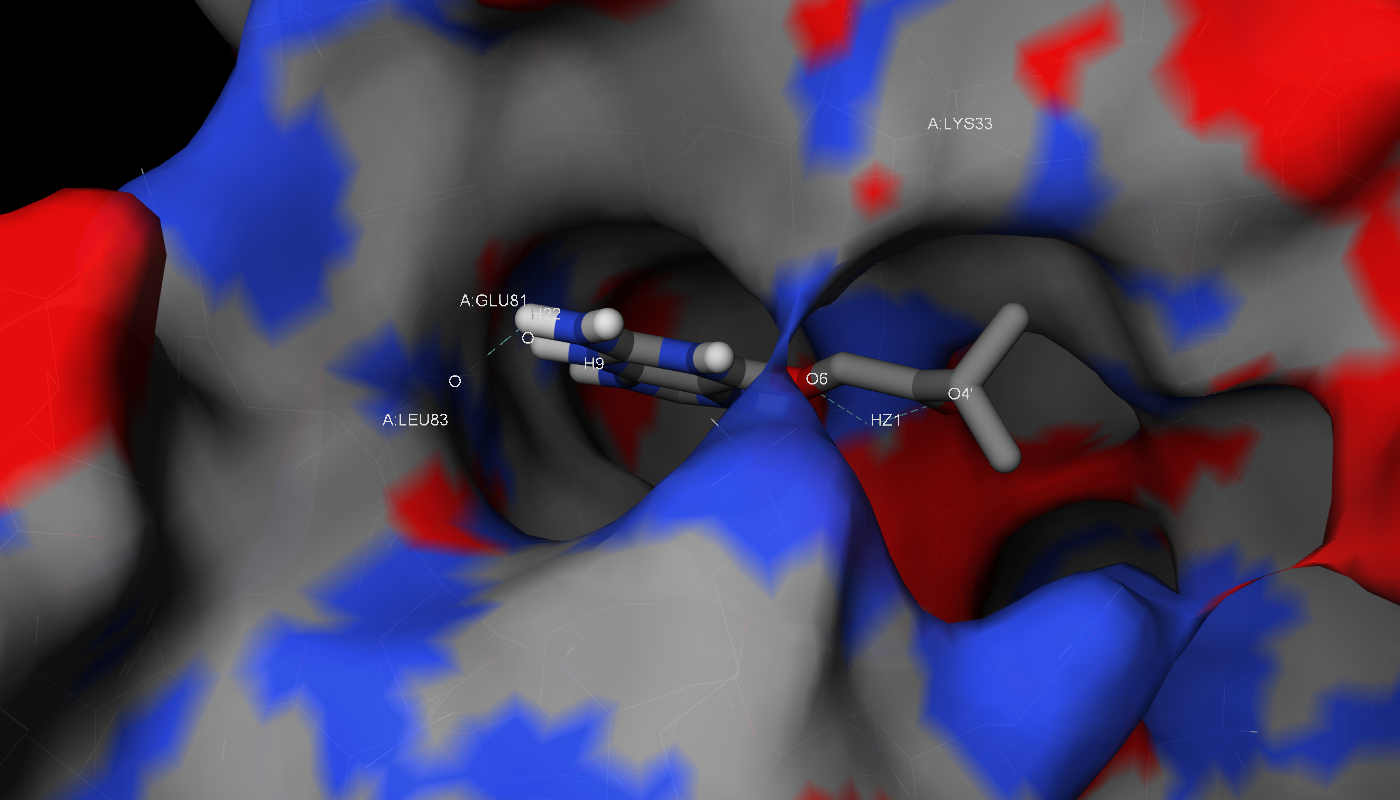

Supplement: S4 Fig — CDK2 is rendered as molecular surface colored by atom type, with an opacity of 0.9 to show the underlying atoms. MBP is rendered as sticks colored by atom type. This figure was created by iview [16]. (PNG) [file pone.0132072.s008.png]

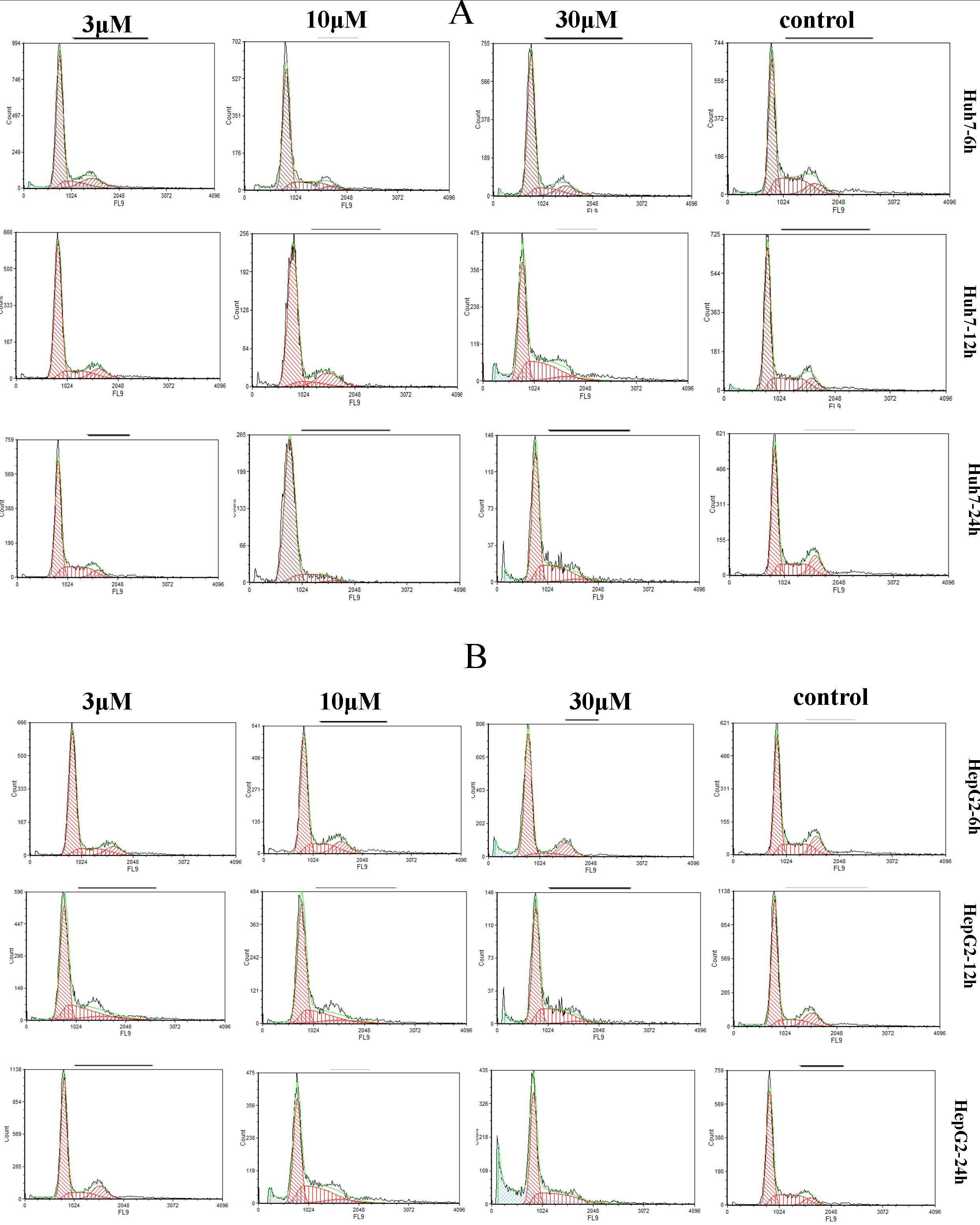

Supplement: S5 Fig — (JPG) [file pone.0132072.s010.jpg]

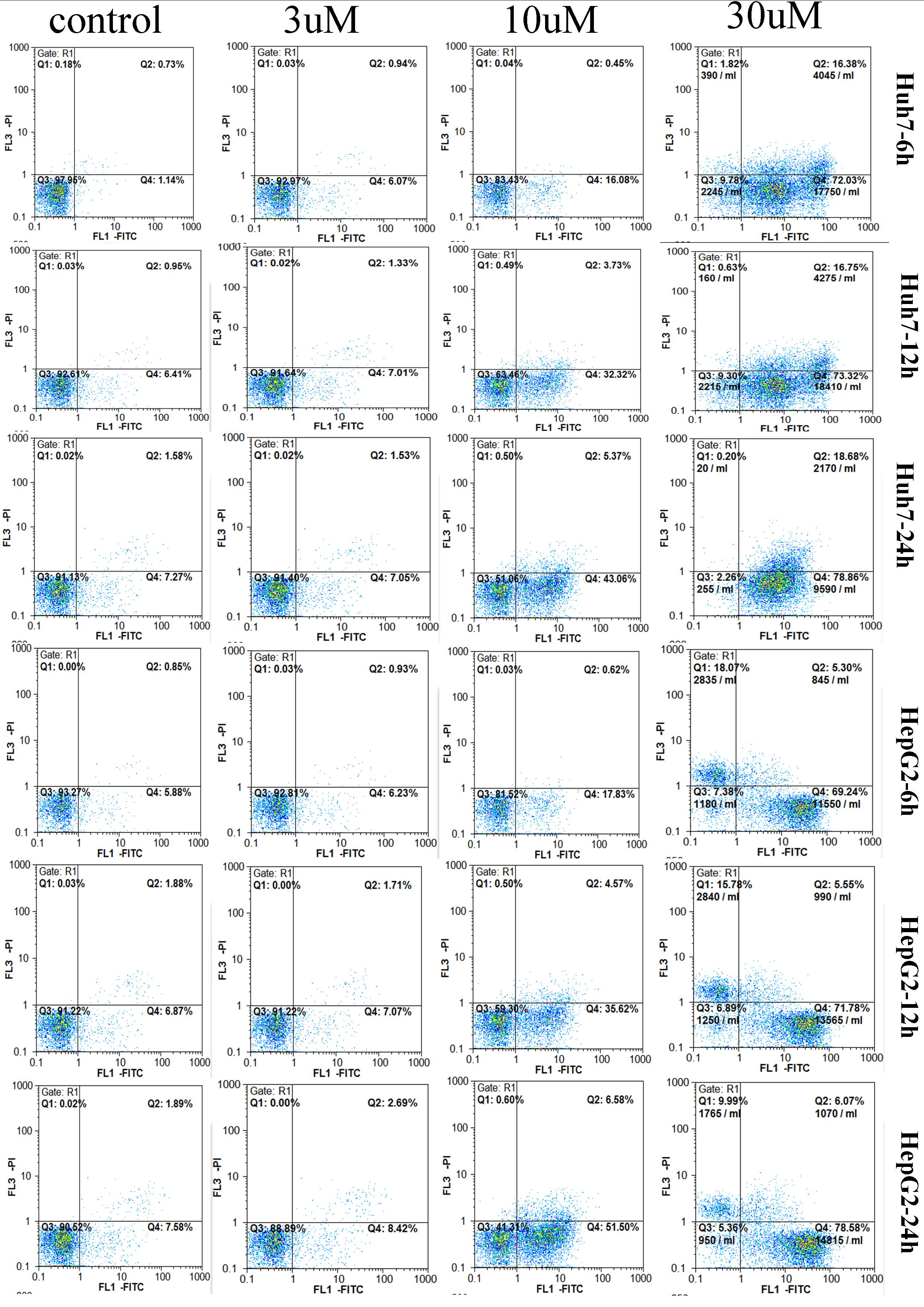

Supplement: S6 Fig — (JPG) [file pone.0132072.s011.jpg]
