# Supplementary material for: In Silico Identification and In Vitro and In Vivo Validation of Anti-Psychotic Drug Fluspirilene as a Potential CDK2 Inhibitor and a Candidate Anti-Cancer Drug
Source: PLoS One. 2015 Jul 6;10(7):e0132072. doi: 10.1371/journal.pone.0132072 (PMC4493148; doi:10.1371/journal.pone.0132072)
Supplement: S4 Table — The table comprises the ZINC ID, the average score, scientific name, clinical uses and references. (PDF) [file pone.0132072.s005.pdf]

Table S3. Details of the nine top-scoring compounds purchased. The idock score is an estimation of binding free energy in kcal/mol units. A more negative value implies a higher predicted binding affinity.

ZINC ID	idock score (kca/mol)	name	clinical uses	references
06716957	-10.46	nilotinib	chronic myeloid leukemia	1
03830332	-10.43	LS-194959	food, drug additive	2
03830768	-10.23	estradiol benzoate	estrogen	3
03881613	-10.08	nandrolone phenylpropionat e	osteoporosis	4
01542113	-10.06	vilazodone	major depressive disorder	5
00537755	-10.02	fluspirilene	chronic schizophrenia	6
00897240	-10.01	azelastine hydrochloride	seasonal allergic rhinitis and perennial allergic rhinitis	7
33974796	-9.98	latuda	schizophrenia	8
01481956	-9.95	paliperidone	schizophreni	9

References

[1] Ellen Weisberg, Paul W. Manley, Werner Breitenstein, Josef Brüggen, Sandra W. Cowan-Jacob, Arghya Ray, Brian Huntly, Doriano Fabbro, Gabriele Fendrich, Elizabeth Hall-Meyers, Andrew L. Kung, Jürgen Mestan, George Q. Daley, Linda Callahan, Laurie Catley, Cara Cavazza, Azam Mohammed, Donna Neuberg, Renee D. Wright, D. Gary Gilliland and James D. Griffin. Characterization of AMN107, a selective inhibitor of native and mutant Bcr-Abl, *Cancer Cell*, 7(2):129-141, 2005.

[2] E. N. Abrahart. Dyes and Their Intermediates. Pergamon Press, Oxford, 1968.

[3] Maurice Fremont-Smith, Joe V. Meigs, Ruth M. Graham and Helen H. Gilbert. Cancer of endometrium and prolonged estrogen therapy. *Journal of the American Medical Association*, 131(10):805-808, 1946.

[4] G. A. Overbeek and J. de Visser. A comparison of the myotrophic and androgenic activities of the phenylpropionates and decanoates of testosterone and nandrolone. *Acta Endocrinologica*, 38(2):285-292, 1961.
[5] Gerd D Bartoszyk, Rainer Hegenbart and Herbert Ziegler. EMD 68843, a serotonin reuptake inhibitor with selective presynaptic 5-HT_{1A} receptor agonistic properties. *European Journal of Pharmacology*, 322(2-3):147-153, 1997.
[6] G. Chouinard, T. A. Ban, H. E. Lehmann and J. V. Ananth. *Current Therapeutic Research, Clinical and Experimental*, 12(9):604-608, 1970.
[7] K. Tasaka and M. Akagi. Anti-allergic properties of a new histamine antagonist, 4-(p-chlorobenzyl)-2-[N-methyl-perhydroazepinyl-(4)]-1-(2H)-phthalazinone hydrochloride (azelastine). *Arzneimittel-Forschung*, 29(3):488-493, 1978.
[8] Takeo Ishiyama, Kumiko Tokuda, Tadashi Ishibashi, Akira Ito, Satoko Toma and Yukihiro Ohno. Lurasidone (SM-13496), a novel atypical antipsychotic drug, reverses MK-801-induced impairment of learning and memory in the rat passive-avoidance test. *European Journal of Pharmacology*, 572(2-3):160-170, 2007.
[9] Michelle Kramer, George Simpson, Valentinas Maciulis, Stuart Kushner, Ujjwala Vijapurkar, Pilar Lim and Mariëlle Eerdekens. Paliperidone Extended-Release Tablets for Prevention of Symptom Recurrence in Patients With Schizophrenia: A Randomized, Double-Blind, Placebo-Controlled Study. *Journal of Clinical Psychopharmacology*, 27(1):6-14, 2007.
