# Supplementary material for: In Silico Identification and In Vitro and In Vivo Validation of Anti-Psychotic Drug Fluspirilene as a Potential CDK2 Inhibitor and a Candidate Anti-Cancer Drug
Source: PLoS One. 2015 Jul 6;10(7):e0132072. doi: 10.1371/journal.pone.0132072 (PMC4493148; doi:10.1371/journal.pone.0132072)
Supplement: S5 Table — (PDF) [file pone.0132072.s009.pdf]

Table S9. The sub-G1 percentages in cell cycle.

	control (%)	3μM (%)	10μM (%)	30μM (%)
Huh7-6h	0	0	0	0
Huh7-12h	0	0	2	9.05
Huh7-24h	0	0	3.1	13.76
HepG2-6h	0	0	0	4.83
HepG2-12h	0	0	0	11.54
HepG2-24h	0	0	4.79	22.4
